# Supplementary material for: JAK/STAT signaling in Drosophila muscles controls the cellular immune response against parasitoid infection
Source: EMBO Rep. 2015 Sep 27;16(12):1664–72. doi: 10.15252/embr.201540277 (PMC4687419; doi:10.15252/embr.201540277)
Supplement: Supplementary file 1 — Appendix [file EMBR-16-1664-s001.pdf]

Appendix

Contents

Appendix Supplementary Methods p. 1
Appendix Table p. 1
Appendix Figures
Figure S1 p. 2
Figure S2 p. 2
Figure S3 p. 3
Figure S4 p. 3

Appendix Supplementary Methods

Mashed-potato fly food

This recipe was developed by members at the former Department of Genetics, Umeå University.

5.5 l water
200 g mashed-potato powder
50 g agar
250 ml treacle
80 g dry yeast
42 ml nipagin solution (dissolve 100 g nipagin in 900 ml 95% ethanol)
7 ml ascorbic acid powder

Boil the water. Add mashed-potato powder, agar, yeast and treacle. Whisk vigorously, reduce the heating, and boil for 15 minutes. Cool to 60°C, add nipagin and ascorbic acid.

Appendix Table

Table S1. Primer sequences used for quantitative PCR.

Gene	Sequence	Reaction efficiency
Rpl32	TTCTGCATGAGCAGGACCTC<br>GGTTACGGATCGAACAAGCG	89%
upd1 (os)	TTCGACTGGCGCTTTCCACGTC<br>CGCAGCTCCACCTTGAATGGCA	96%
upd2	GATCCGTTGGCTGGCGTGTGAA<br>TCAACGAGGCGGTCACCAAGGA	103%
upd3	GCCGCGATATAAAGATACAAAGATA<br>ACTTTGCTTTTGTAACGCTGTTAGT	83%
Socs36E	GAGGCCGAAGTCGTCG<br>GAGGATGTTGCTGCTCCC	83%

Appendix Figures

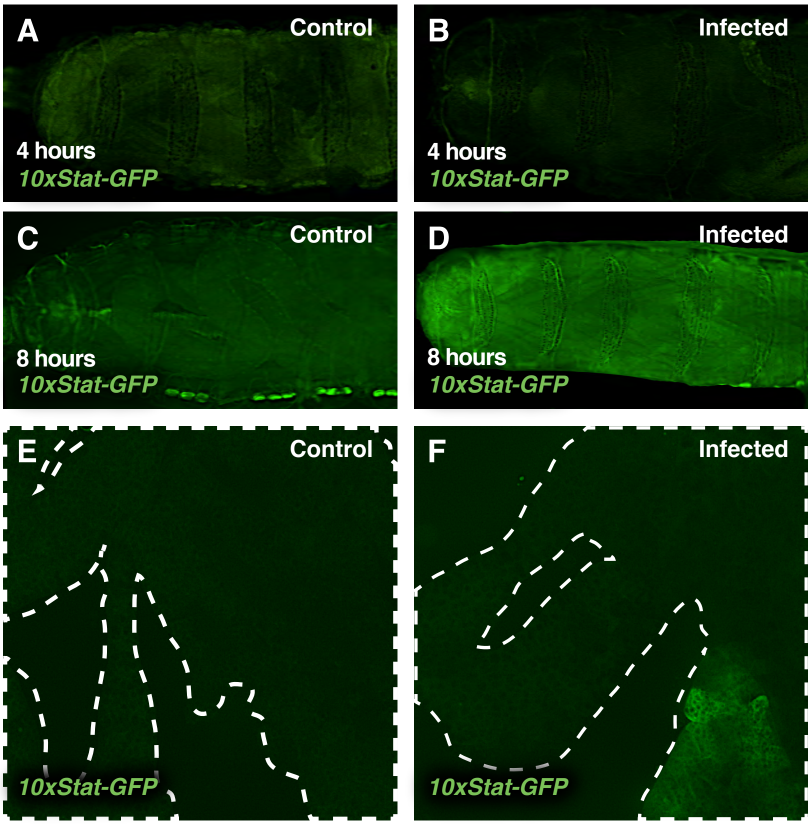

Figure S1. Induction of JAK/STAT activity in larval muscles and fat body after wasp infection.

A–D JAK/STAT signaling, visualized with the *10XStat-GFP* reporter, in control (A, C) and infected (B, D) larvae at 4 hours (A, B) and 8 hours (C, D) after the time of infection.

E, F JAK/STAT signaling in fat body reported by *10XStat-GFP* fly strain in control (E) and after 27 hours wasp infection (F). Dashed lines outline the fat body tissue.

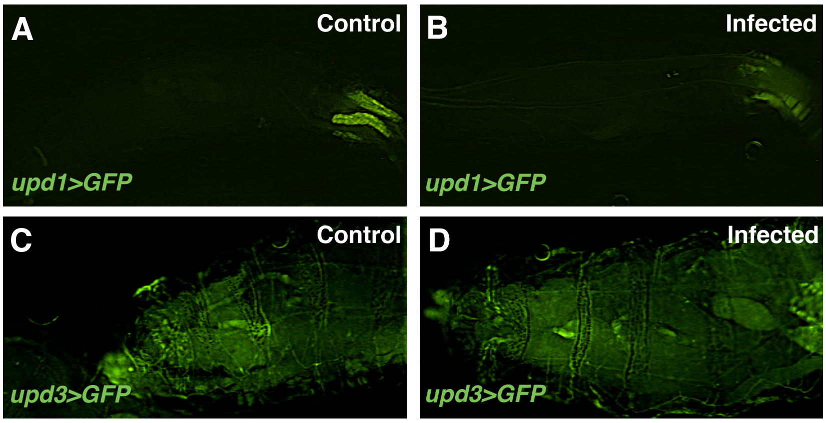

Figure S2. Overview of *upd1* and *upd3* expression pattern after wasp infection.

A, B *upd1* expression pattern reported by *upd1>GFP* in control larvae (A) and in wasp-infected larvae 27 hours after infection (B).

C, D *upd3* expression pattern reported by *upd3>GFP* in control larvae (C) and in wasp-infected larvae 27 hours after infection (D).

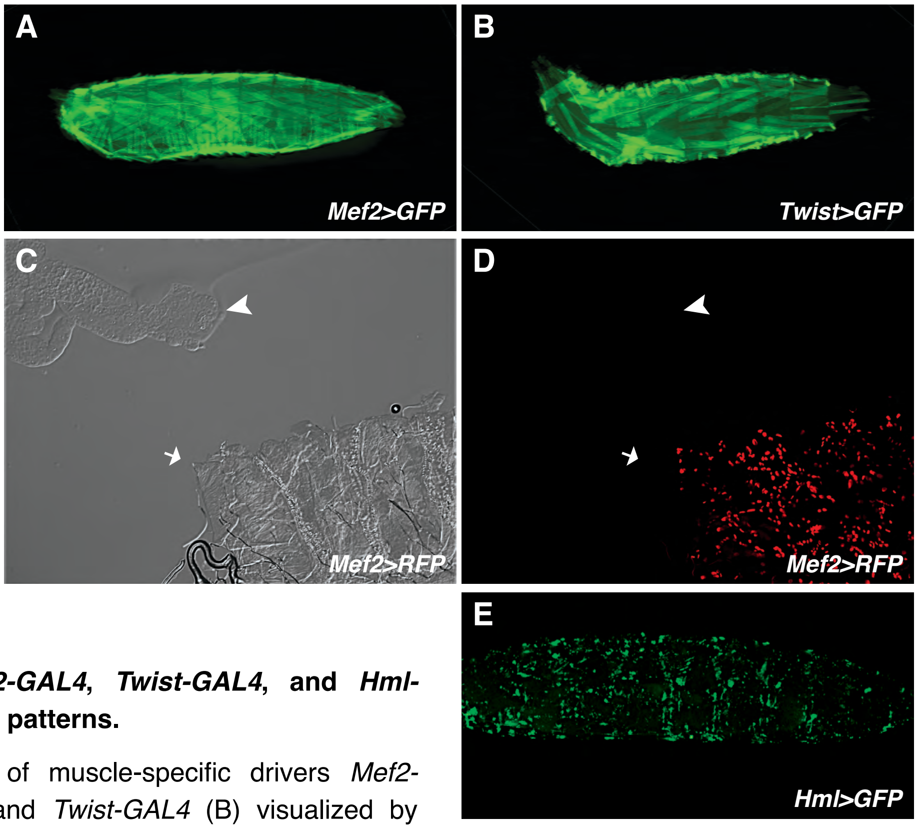

Figure S3. *Mef2-GAL4*, *Twist-GAL4*, and *Hml-GAL4* expression patterns.

A, B Expression of muscle-specific drivers *Mef2-GAL4* (A) and *Twist-GAL4* (B) visualized by *GFP*.

C, D Bright-field (C) and fluorescence images (D) of Mef2-GAL4 driven RFP showing expression in larval muscles (arrows), but not in fat body (arrowheads).

E Expression of the hemocyte-specific driver *Hml-GAL4 > GFP*, showing the characteristic bands of sessile hemocytes.

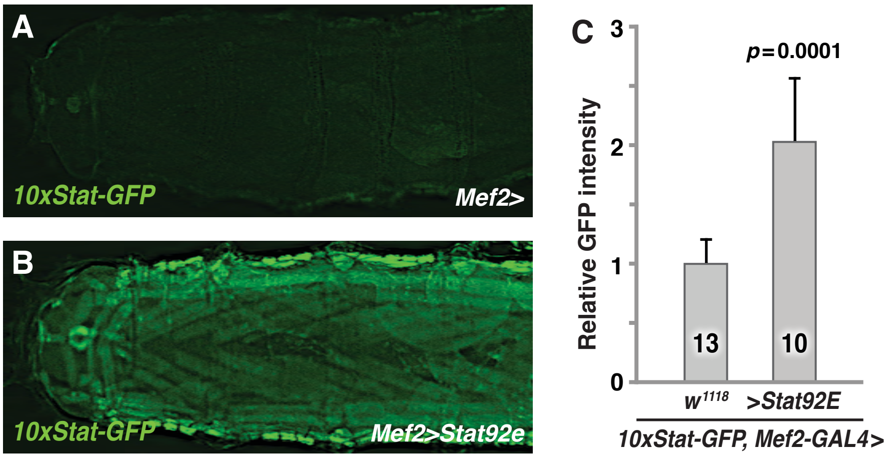

Figure S4. Activation of JAK/STAT signaling in muscles by overexpression of wild type *Stat92E*.

A, B Activation of the *10xStat-GFP* reporter in muscles by expression of wild type *Stat92E* with the *Mef2-GAL4* driver (B), compared with the driver-only control (A).

C Quantification of GFP signal in muscles from the indicated total number of larvae. Bars show average and standard deviation (Unpaired t test).
